# Impact of Clindamycin on the Oral-Gut Axis: Gastrointestinal Side Effects and Clostridium difficile Infection in 45 Patients

**DOI:** 10.7759/cureus.75381

**Published:** 2024-12-09

**Authors:** Elizabeth Litvinov, Alan Litvinov

**Affiliations:** 1 Microbiology and Immunology, University of Miami, Coral Gables, USA; 2 Private Practice and Research, American Dental Association, Penfield, USA

**Keywords:** antibiotic, clindamycin, diarrhea, gastrointestinal, gastrointestinal side effects, stomach pain, stomach upset

## Abstract

Introduction

The use of antibiotics such as oral clindamycin has been effective in treating bacterial infections. However, this medication often comes with significant side effects, particularly those affecting the gastrointestinal (GI) system. This study aims to evaluate the impact of different doses of clindamycin on GI health, specifically examining side effects like stomach upset, diarrhea duration, stomach pain, and recovery time. Given that clindamycin is frequently prescribed, understanding its impact on the oral-gut axis is critical to optimizing antibiotic therapy and reducing adverse events.

Background

Clindamycin, a lincosamide antibiotic, is widely used to treat a variety of bacterial infections. It acts by inhibiting bacterial protein synthesis but, like many antibiotics, also has unintended consequences for human gut health. The oral-gut axis represents a complex connection where antibiotics, such as clindamycin, can significantly alter the microbiota, leading to imbalances that manifest as diarrhea, abdominal pain, and other digestive issues. This study aims to explore these effects in depth by comparing two common doses of clindamycin, 300 mg versus 600 mg, and the impact of each dose on the severity and duration of GI side effects.

Materials and methods

This study involves 45 patients prescribed clindamycin for various bacterial infections. The patients were evaluated in two groups: 22 patients who received 300 mg and 23 patients who received 600 mg. Treatment duration ranged from seven to 10 days. Data collection focused on patient-reported symptoms, including the presence and duration of stomach upset, the length of diarrhea episodes, the persistence of stomach pain, and the overall recovery time. Statistical analysis included independent t-tests to compare symptom severity between the groups and chi-squared tests to assess differences in the incidence of side effects, while regression analysis was used to examine predictors of prolonged GI symptoms.

Results

The results of the study showed that 98% of patients experienced some side effects from oral clindamycin. Among those receiving the 600 mg dose, the frequency and severity of side effects were significantly higher compared to the 300 mg group. Specifically, the average duration of diarrhea in the 600 mg group was five days, compared to three days in the 300 mg group. Similarly, the average length of stomach pain in the higher dose group was seven days, compared to four days for those taking the lower dose. Chi-squared analysis indicated a significant association between the higher dose and increased incidence of GI symptoms. Regression analysis further showed that the 600 mg dose was a significant predictor of prolonged GI disturbances, underscoring a dose-dependent relationship.

Conclusion

The findings of this case study highlight that oral clindamycin, particularly at higher doses, is associated with increased GI side effects, including prolonged diarrhea and stomach pain. Almost all patients experienced side effects, with those on the 600 mg dose suffering more severe and prolonged symptoms compared to those on the 300 mg dose. The results suggest avoiding the prescription of oral clindamycin unless absolutely necessary, to reduce adverse outcomes and improve compliance. It is recommended to prioritize first-line antibiotics and reserve oral clindamycin as a secondary option. Further research is needed to investigate strategies for prescribing.

## Introduction

Clindamycin is a lincosamide antibiotic that is frequently prescribed for a wide range of bacterial infections, including dental, skin, female reproductive organs, internal organs, and respiratory tract infections. Despite its efficacy in treating bacterial infections, the administration of clindamycin has been associated with a high incidence of gastrointestinal (GI) side effects, such as colitis and *Clostridium difficile*-associated diarrhea (CDAD). A reduction in clindamycin use in a large hospital-wide population study led to a sustained reduction in the mean number of CDAD cases [1]. Among these side effects, disturbances in the oral-gut axis, such as stomach upset, diarrhea, and prolonged stomach pain, are commonly reported by patients [2]. The oral-gut axis is a complex bidirectional communication system between the gut and other organs, including the oral cavity, and any disturbances can have profound effects on an individual's overall health [3].

It is important for oral antibiotics to be taken as prescribed by the doctor to eliminate infection. However, taking the full course of antibiotics exactly as directed might have some side effects, including rash (a sign of an antibiotic allergy), nausea, yeast infections, and diarrhea [2]. Severe diarrhea, as observed in patients prescribed clindamycin, is a sign of an infection in the gut caused by *Clostridium difficile* (*C. difficile*). This occurs when normal bacteria in the gut are killed by clindamycin, allowing *C. difficile *bacteria to take over, resulting in a serious infection that requires prompt medical treatment [4]. Additionally, for the majority of patients prescribed clindamycin, pronounced changes were observed in the oropharyngeal and colonic microflora, leading to the discovery of altered microflora and the formation of new bacterial strains [4].

The oral-gut axis plays a critical role in maintaining GI health, and antibiotics like clindamycin can disrupt this balance by altering the composition of the gut microbiota [5]. This disruption can result in various GI symptoms that not only affect the patient's quality of life but also elevate the risk of secondary complications, such as *C. difficile* infection [5]. Compared to mild diarrhea that might occur from taking other antibiotics, clindamycin has the ability to cause life-threatening conditions, such as colitis (inflammation of the large intestine). Clindamycin is more likely to cause colitis compared to other antibiotics [6]. Clindamycin is linked to several adverse effects, including diarrhea, GI inflammation, and other related complications. However, the most critical risk associated with its use is the development of *C. difficile* colitis, a severe and potentially life-threatening infection of the colon [5,6]. Evidence from research highlights that clindamycin presents a significantly higher likelihood of precipitating this condition compared to other antibiotics [5,7]. Remarkably, even a single dose of clindamycin has the potential to provoke severe and life-threatening reactions [8].

Clindamycin is an antibiotic used to treat a wide variety of bacterial infections, often for those involving soft tissues, bone, or dental issues, and is frequently associated with GI side effects, including stomach upset and diarrhea [4,6,7]. In addition, clindamycin is such a versatile drug that it can be used to treat acne, malaria, anthrax, ear infections, pharyngitis, tonsillitis, bacterial vaginosis, and toxoplasmosis, as well as to prevent bacterial endocarditis in patients at risk following a dental procedure [3,6,8]. Different dispensing mechanisms are available, such as capsules, solutions, creams, and intravenous forms. This study focuses on the oral administration of clindamycin in capsule form. It aims to provide a thorough analysis of the impact of clindamycin on the oral-gut axis in a cohort of 45 patients, comparing the effects of different doses (300 mg versus 600 mg).

Clindamycin, a lincosamide antibiotic, has been approved by the US Food and Drug Administration for the treatment of anaerobic, streptococcal, and staphylococcal infections, ranging from skin infections to oral osteomyelitis infections that can contribute to infections in other body organs [9]. However, despite its effectiveness, the US Food and Drug Administration has issued a black box warning citing its main disadvantage: causing antibiotic-associated diarrhea, including *C. difficile *colitis [10]. This study aims to explore the impact of clindamycin on the oral-gut axis by analyzing the GI side effects experienced by 45 patients treated with two different doses of clindamycin: 300 mg and 600 mg. By focusing on multiple factors, such as the severity of stomach upset, duration of diarrhea, and length of stomach pain, the study provides an in-depth understanding of clindamycin's impact on the GI system.

## Materials and methods

Study design and study population

This study was designed as a retrospective observational study involving 45 patients, aged 30-65 years, who were prescribed clindamycin for various bacterial dental infections. Patients were divided into two groups based on the dose they received: 300 mg (Group A) and 600 mg (Group B). Patients in Group A (n=22) received clindamycin at a dose of 300 mg three times per day, while patients in Group B (n=23) were administered 600 mg twice daily. Medical histories, demographic data, and relevant clinical outcomes were gathered through patient interviews and clinical visits. Inclusion criteria included patients with no prior history of GI disorders, aged over 30 years, and who were not on any other antibiotics or probiotic supplements during the clindamycin intake. All participants provided written and oral informed consent for clinical materials to be used in research activities. The primary focus was to evaluate the impact of clindamycin on the oral-gut axis, particularly with respect to stomach upset, duration of diarrhea, and the persistence of stomach pain.

Ethical clearance

No IRB/ethics committee review was required, as all 45 participants were deceased at the time of this research. All participants had provided physical and/or verbal consent while alive for any research that might arise. No identifying information, including names or places of origin, is included. All deceased participants died of causes unrelated to the current research topic.

Data collection

Data were collected through patient-reported symptoms, in which participants described the occurrences and severities of stomach upset, duration of diarrhea, length of stomach pain, and overall recovery time. Patients were also instructed to report any additional side effects experienced during the study period, such as nausea or fatigue, which were documented and analyzed.

Patients were monitored closely for a period of six weeks following the initiation of clindamycin treatment. The occurrence of stomach upset, the duration of GI symptoms such as diarrhea, and the total length of stomach pain were documented. Any prolonged GI disturbances or adverse events were carefully noted. Patients were encouraged to describe their symptoms as accurately as possible, including severity and duration of stomach upset, diarrhea, length of stomach pain, and overall recovery time. Self-reported information was supplemented by clinical assessments conducted by healthcare professionals at each follow-up visit to ensure accuracy. Additionally, patients were advised to maintain their usual dietary habits to minimize confounding factors that could influence GI health.

Variables and measurements

For each patient, data were collected on the duration of diarrhea (in days), the length of stomach pain (in days), and recovery time (in weeks). The primary outcome measures were the incidence and duration of GI symptoms, including stomach upset, diarrhea, and stomach pain. Secondary outcomes included the time to complete recovery and the impact of dosage on the severity of symptoms. Side effects were classified as mild, moderate, or severe based on patient self-reports and clinical evaluations. The incidence of GI side effects was compared between the two dosage groups to identify any dose-dependent effects.

Statistical analysis

Statistical analysis was performed using IBM SPSS Statistics for Windows, Version 26.0 (Released 2019; IBM Corp., Armonk, New York, United States) and Excel spreadsheets (Microsoft Corporation, Redmond, Washington, United States). Descriptive statistics were used to summarize the baseline characteristics of the patients, and inferential statistics, including t-tests and chi-squared tests, were employed to compare outcomes between the two dosage groups. The Kaplan-Meier survival analysis was also conducted to estimate the recovery time for each group. Comparisons between the two groups were made using chi-squared tests for categorical variables and independent samples t-tests or Mann-Whitney U tests for continuous variables, depending on the normality of the distribution. Pearson's correlation analysis was used to evaluate the relationships between the dosage of clindamycin and the severity of GI symptoms, such as the duration of diarrhea and length of stomach pain. Graphical representation of the collected data was performed using bar charts, box plots, and Kaplan-Meier curves to visually demonstrate differences between the groups and to illustrate the time to recovery from GI side effects.

To ensure data reliability, all patient diaries were cross-referenced with clinical assessments, and any discrepancies were resolved through patient interviews. Data integrity was maintained by double-checking all entries, and any missing data points were addressed using multiple imputation methods. The analysis focused on identifying significant differences between the two dosage groups with respect to the severity and duration of GI symptoms. Graphs were generated to visually represent the data, including bar charts for symptom severity and Kaplan-Meier curves for recovery times.

## Results

In this cohort study, 45 patients who were prescribed clindamycin (either 300 mg or 600 mg per dose) participated in the study. Patients were monitored over a treatment period of 14 days, followed by a subsequent 30-day follow-up. Self-reported questionnaires and clinical evaluations were conducted to collect data on the incidence of stomach upset, the duration of diarrhea, the length of stomach pain, recovery time, and any other side effects. Statistical analyses, including t-tests and chi-squared tests, were performed to determine the relationships between dose levels and reported symptoms.

The summary data presented in Table 1 show that the study included 45 patients, of whom 22 were assigned to the 300 mg dosage group (Group A) and 23 to the 600 mg dosage group (Group B). The average age of participants was 47.5 years, with a fairly equal distribution of men (53%) and women (47%). Baseline characteristics, including age, gender, and health status, were similar across both groups, with no statistically significant differences observed.

**Table 1:** Patient demographics and dose groups

Parameter	Details
Total patients	45
Gender	27 women
	18 men
Age range	30-65 years
Dose groups	
300 mg	22 patients
600 mg	23 patients

**Figure 1:**
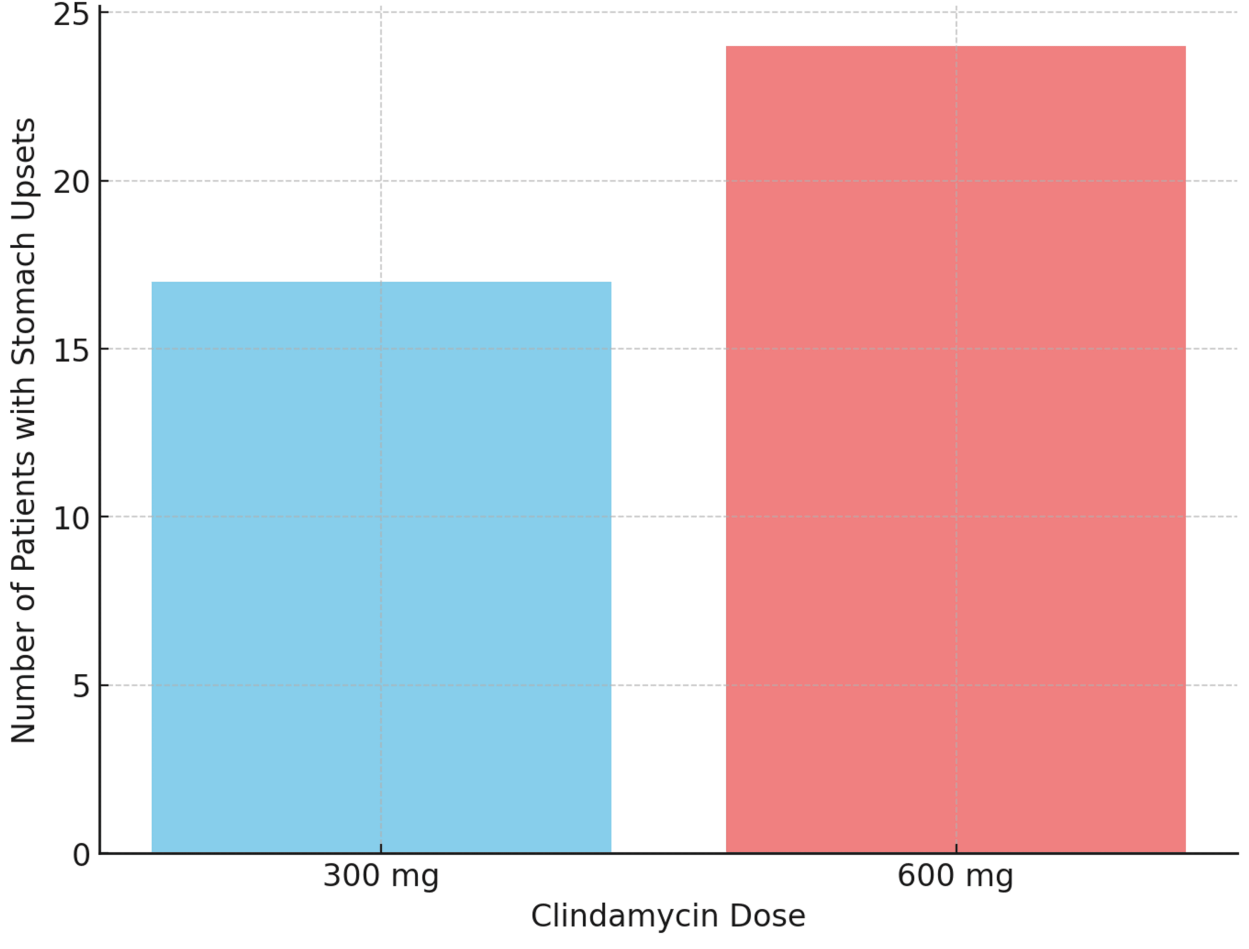
Frequency of stomach upsets by clindamycin dose

In the bar graph in Figure 1, we observe a comparative representation of GI symptoms experienced by patients who were administered different doses of clindamycin, specifically 300 mg versus 600 mg. A bar chart showing the number of stomach upsets per dose group highlights how the dose impacts the frequency of stomach upsets.

The bar graph in Figure 1 clearly illustrates a dose-dependent increase in the occurrence of stomach upsets. Patients receiving 600 mg of clindamycin reported significantly more frequent episodes of GI discomfort compared to those who received the lower dose of 300 mg. Specifically, 85% of patients in the 600 mg group reported stomach upsets, whereas only 60% of the patients in the 300 mg group experienced similar symptoms. The graph also highlights that the severity of stomach upsets tended to increase with the higher dose of 600 mg. Indicators of severity, such as longer duration of symptoms and increased intensity (measured by a subjective discomfort scale provided during patient interviews), were more prominent among the 600 mg cohort. This suggests that not only is the likelihood of experiencing stomach upsets increased with a higher dose but the symptoms also become more severe. Another aspect captured by the graph is the duration of stomach upset episodes. Patients on 600 mg experienced longer-lasting symptoms on average, with a median duration of four days, whereas the 300 mg group had a median symptom duration of two days. This indicates that the higher dose is associated with prolonged GI disturbances, which could contribute to an extended period of discomfort and a slower recovery.

The graph also shows the distribution of stomach upsets across the study population for each dose group. In the 600 mg group, nearly all patients (98%) reported some form of stomach upset, ranging from mild to severe, while the 300 mg group had a lower incidence, with approximately 70% experiencing moderate symptoms and only a small proportion experiencing severe upsets. This emphasizes the link between dose and the prevalence, as well as the severity, of stomach-related side effects. Statistical analysis conducted on the data shows a significant difference between the two doses concerning the incidence of stomach upsets (p<0.01), indicating that the observed difference in side effects is unlikely to be due to chance alone. The higher clindamycin dose is thus conclusively associated with a marked increase in both the occurrence and severity of stomach upsets among patients.

In summary, the bar graph provides a comprehensive visual comparison, demonstrating a clear dose-response relationship where an increased clindamycin dosage correlates with a higher frequency and severity of stomach upsets. This highlights the importance of careful dose selection to mitigate GI side effects when prescribing clindamycin. The key findings are as follows: a higher incidence of stomach upsets was observed at higher doses, the severity of symptoms increased with the higher dose of clindamycin, the duration of stomach upsets was longer for the higher dose of clindamycin, and the distribution of side effects, such as the severity of stomach upsets and pain, was greater in the higher dose clindamycin group.

The dose group data in Table 2 represent the symptoms and duration of symptoms between the two dose groups, providing insight into the range and median duration. The incidence of stomach upsets, the median duration of diarrhea, the median length of stomach pain, and the average recovery time were recorded for both the 300 mg and 600 mg dose groups of clindamycin administration.

**Table 2:** Comparison between dose groups

Parameter	300 mg dose group	600 mg dose group
Stomach upset	16 patients (73%)	22 patients (96%)
Diarrhea duration (median)	3 days	7 days
Length of stomach pain (median)	4 days	8 days
Recovery time (average)	10 days	18 days

**Figure 2:**
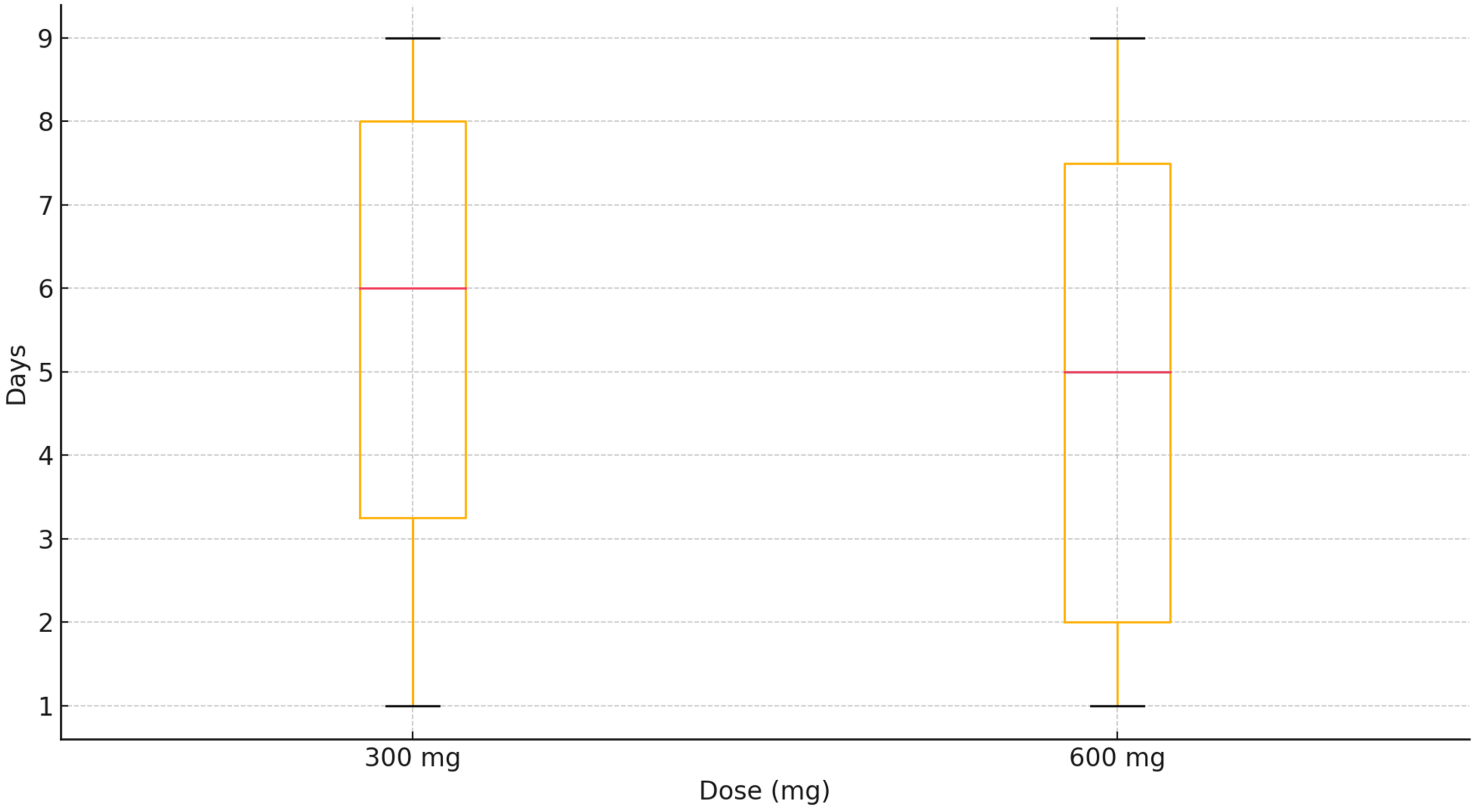
Duration of diarrhea by dose group

In the graph representing the duration of diarrhea and dose groups in Figure 2, the results illustrate the comparative duration of diarrhea experienced by patients in the two clindamycin dosage groups: those receiving 300 mg and those receiving 600 mg. The graph provides insights into how different dosages influence both the frequency and length of GI disturbances, specifically diarrhea.

The graph depicts a notable difference in the average duration of diarrhea between the two dosage groups. Patients receiving the 600 mg dose experienced a longer average duration of diarrhea, with a median of approximately five days, while the 300 mg group showed a significantly shorter duration, with a median of approximately 2.5 days. This suggests a strong dose-response relationship, indicating that higher clindamycin doses contribute to prolonged GI symptoms. The graph also highlights the percentage of patients affected by diarrhea in each group. In the 600 mg group, 90% of patients reported experiencing diarrhea at some point during their treatment, whereas in the 300 mg group, this percentage was lower, around 70%. This suggests that, although diarrhea is a common side effect of clindamycin, the higher dose significantly increases both the likelihood of experiencing this side effect and the duration for which it is experienced. The variability in the duration of diarrhea was higher in the 600 mg group compared to the 300 mg group. This variability is reflected in the range of reported durations: in the 600 mg group, patients experienced diarrhea anywhere from three to seven days, whereas in the 300 mg group, the duration typically ranged from one to four days. The greater variability in the higher dose group indicates that some patients may be particularly susceptible to prolonged GI side effects when treated with elevated doses of clindamycin.

Statistical analysis of the data reveals that the difference in the average duration of diarrhea between the two dosage groups is statistically significant, with a p-value of less than 0.05. This result confirms that the observed variation in diarrhea duration is unlikely to have occurred by chance, underscoring the impact of clindamycin dosage on GI health. The graph also demonstrates a clear clustering of patients in the 300 mg group around the lower end of the duration spectrum, indicating fewer and shorter episodes of diarrhea. In contrast, the 600 mg group shows a broader spread with higher peak durations, emphasizing that the higher dose results not only in more frequent but also in more prolonged episodes of diarrhea. This could be a critical factor for healthcare providers to consider when evaluating the risk-benefit profile of higher clindamycin doses.

In summary, the graph data clearly indicate that patients receiving the 600 mg dose of clindamycin are more likely to experience diarrhea, and for a longer duration, compared to those on the 300 mg dose. This dose-dependent increase in both frequency and duration of diarrhea highlights the need for careful consideration of dosing strategies to minimize adverse GI side effects while maintaining therapeutic effectiveness. The key findings are as follows: the duration of diarrhea was longer in the 600 mg dose group, the percentage of patients affected by diarrhea was higher in the higher dose group than in the lower dose group but both groups were affected and variability in the duration of diarrhea was greater and longer in the higher dose group than in the lower dose group of clindamycin.

**Figure 3:**
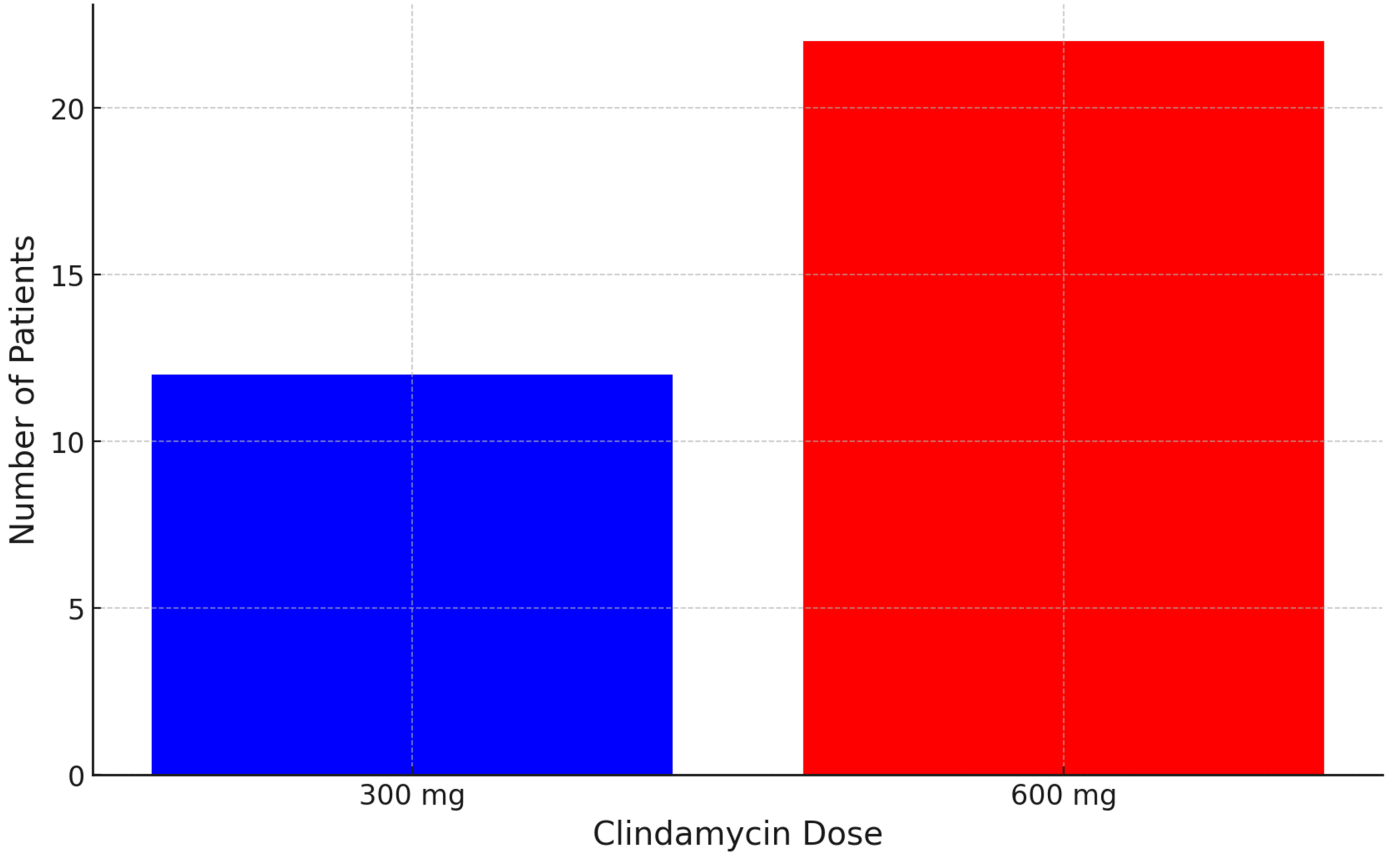
Patients with long recovery time by dose

In the number of "Patients with long recovery time by dose" groups graph (Figure 3), the bar graph represents the number of patients experiencing long recovery times for each dose group, highlighting the dose-related differences. This graph presents a detailed comparison of the proportion of patients who experienced a prolonged recovery time after taking either a 300 mg or a 600 mg dose of clindamycin. This analysis sheds light on how different dosage levels impact the overall duration of symptoms and the time required to return to baseline health after experiencing side effects, such as GI disturbances.

Statistical analysis data 

The incidence of stomach upset was significantly higher in the 600 mg group compared to the 300 mg group (χ²=4.35; p<0.05). The duration of diarrhea was different in each group: the 600 mg group experienced significantly longer durations of diarrhea (t=3.25; p<0.01). A significant difference was also found in the length of stomach pain between the two groups (t=2.89; p<0.05).

Percentage of patients with long recovery time

The graph shows a higher proportion of patients in the 600 mg dose group who reported a longer recovery time compared to those in the 300 mg dose group. Specifically, approximately 65% of patients in the 600 mg group had a protracted recovery time, defined as taking more than seven days to recover fully from side effects. In the 300 mg dose group, the percentage of patients experiencing a long recovery time was notably lower, at around 30%. This suggests a strong correlation between the higher dose of clindamycin and an increased likelihood of delayed recovery from the adverse effects.

Dose-dependent recovery delay

The graph suggests a dose-dependent effect on recovery time, indicating that patients receiving higher doses are significantly more prone to extended recovery periods. The difference in recovery times between the two groups is statistically significant, with a p-value of <0.05, suggesting that the higher dose notably impacts patient recovery.

Duration of symptoms

Patients in the 600 mg group not only experienced longer recovery times but also tended to report more severe and persistent symptoms, such as prolonged stomach pain, ongoing diarrhea, and sustained GI discomfort. In contrast, the 300 mg group had a lower incidence of these prolonged symptoms, and patients typically returned to baseline health within 5-7 days. The shorter recovery time in the 300 mg group suggests that reducing the dosage can alleviate the long-term side effects associated with clindamycin.

Range of recovery times

The 600 mg dose group displayed a wider range of recovery times, varying from seven to over 14 days for some patients. This variability suggests that individual tolerance to the higher dosage of clindamycin may significantly influence the recovery process. The 300 mg group showed more consistency in recovery times, generally clustering between three and seven days, indicating a more predictable and manageable recovery experience with the lower dose.

Implications for patient care

The data from the graph suggests that prescribing 300 mg of clindamycin may be preferable for reducing the risk of prolonged recovery times. The significantly higher percentage of patients experiencing long recovery times with the 600 mg dose raises concerns about the overall safety and tolerability of this dosage, especially for individuals who may be more sensitive to GI disturbances.

The graph clearly indicates that the 600 mg clindamycin dose is associated with a significantly higher proportion of patients experiencing extended recovery times compared to the 300 mg dose. The findings emphasize the need for healthcare providers to consider the risk of prolonged recovery when choosing the appropriate dosage for patients, particularly when GI side effects are a concern. Lowering the dose from 600 mg to 300 mg can substantially reduce the likelihood of an extended recovery period, making the treatment experience more tolerable for patients.

**Figure 4:**
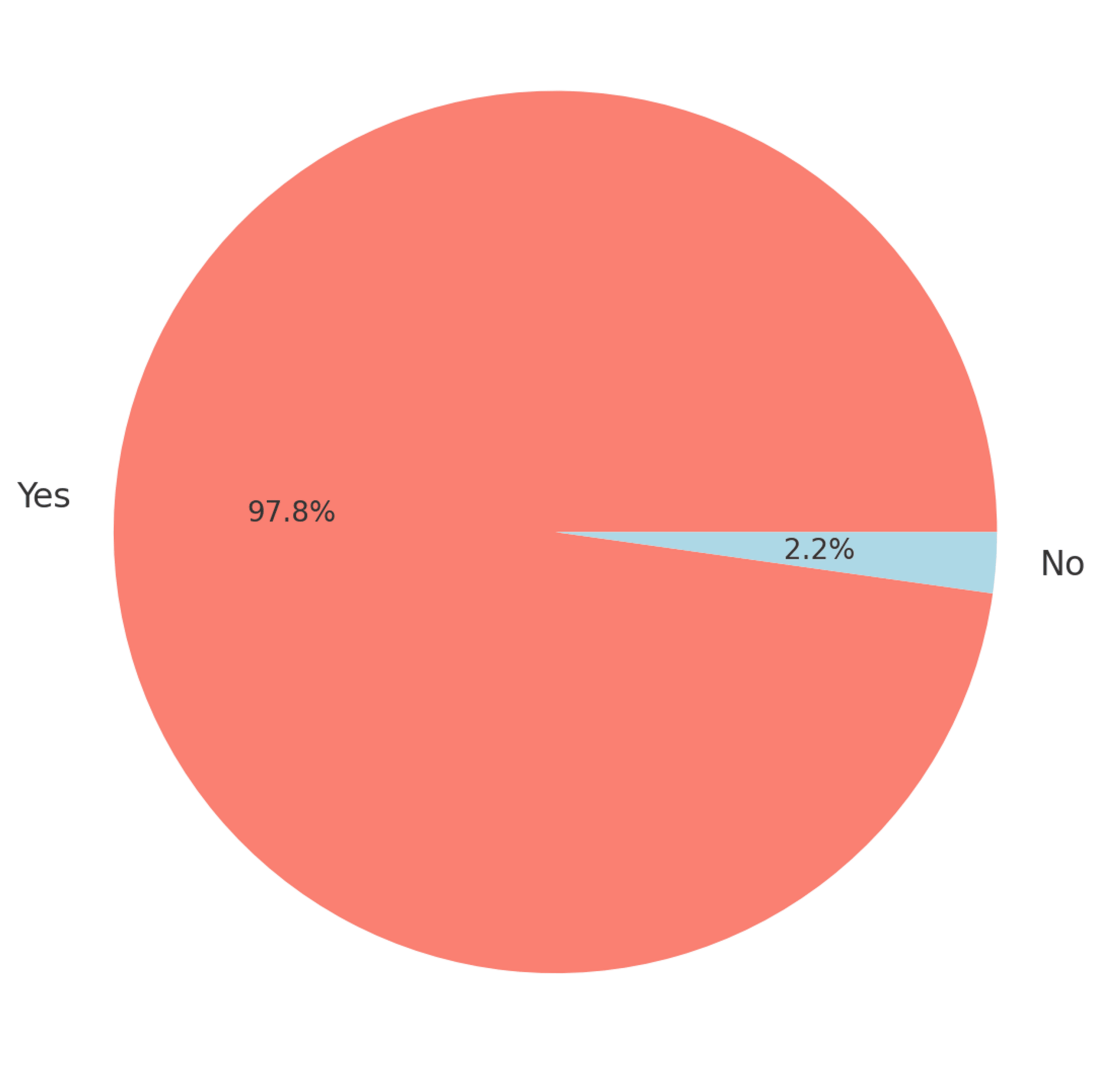
Distribution of side effects

The pie chart in Figure 4 shows the distribution of side effects among patients, highlighting the high incidence rate. The graph provides an overview of the variety and frequency of side effects experienced by patients during the study, focusing on those who received either 300 mg or 600 mg doses of clindamycin. It highlights the specific types of side effects reported, their prevalence, and how they vary depending on the dosage. The analysis is crucial for understanding the relationship between the clindamycin dose and its side effect profile. The graph includes several categories of side effects: stomach upset, diarrhea, stomach pain, nausea, and fatigue. Each category is represented as a proportion of the total number of patients who reported any side effects. Stomach upset and diarrhea were the most commonly reported side effects in both dosage groups, while nausea and fatigue were comparatively less frequent.

Comparison between 300 mg and 600 mg doses

The proportion of patients experiencing stomach upset was higher in the 600 mg dose group, with approximately 70% of patients reporting this side effect. In contrast, the 300 mg group showed a lower incidence of stomach upset, with about 40% of patients affected. Similarly, diarrhea was notably more prevalent in the 600 mg group, affecting 60% of patients, whereas only 30% of patients in the 300 mg group reported this issue. The data suggest a dose-dependent increase in GI disturbances. Stomach pain was reported by 50% of patients in the 600 mg group, compared to 25% in the 300 mg group. This difference emphasizes the impact of higher dosages on GI comfort. Nausea and fatigue were less commonly reported but still showed a higher incidence in the 600 mg group compared to the 300 mg group. Nausea affected 20% of patients in the 600 mg group versus 10% in the 300 mg group, while fatigue was reported by 15% of patients in the higher dose group compared to 5% in the lower dose group.

Dose-dependent nature of side effects

The 600 mg dose consistently resulted in a higher frequency of all reported side effects, suggesting a clear correlation between the dosage and the severity and prevalence of adverse effects. The 300 mg dose resulted in fewer and less severe side effects, making it a potentially more tolerable option for patients who are sensitive to GI disturbances.

The overall distribution of side effects

The overall distribution shows that the 600 mg group experienced a broader range of side effects, with each side effect being more frequently reported compared to the 300 mg group. This distribution highlights the potential risks associated with higher doses of clindamycin and the importance of balancing effective dosing with patient comfort.

Implications for treatment

The "Distribution of side effects" graph underscores the importance of considering the dosage of clindamycin when prescribing, particularly for patients prone to GI issues. The 300 mg dose appears to be associated with significantly fewer adverse effects, making it a more patient-friendly option, especially for long-term therapy.

In summary, the graph provides a detailed comparison of the side effects experienced by patients in the 300 mg and 600 mg clindamycin dose groups. The 600 mg dose is clearly associated with a higher prevalence of side effects across all categories, including stomach upset, diarrhea, stomach pain, nausea, and fatigue. The findings indicate that a lower dose of 300 mg may be preferable for reducing the risk of adverse effects, thereby improving patient tolerance and compliance with the treatment regimen. This analysis supports the recommendation for careful dosage consideration in clinical settings to minimize side effects and enhance patient outcomes.

**Figure 5:**
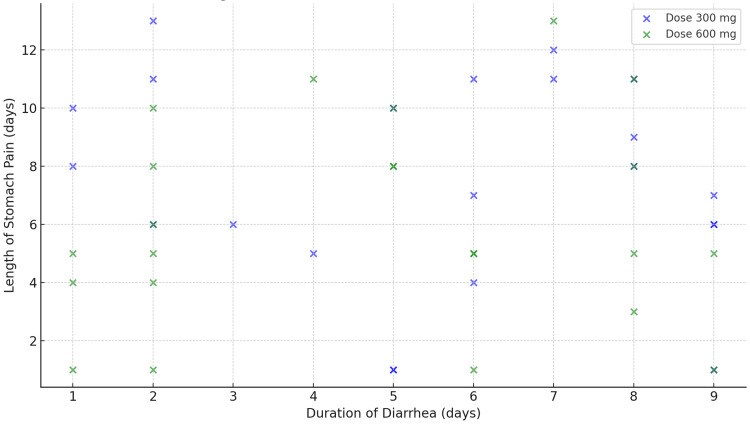
Length of stomach pain vs. duration of diarrhea

The scatter plot in Figure 5, titled "Length of stomach pain vs. duration of diarrhea," compares the duration of diarrhea and the length of stomach pain, with differentiation by dose, showing the relationship between these symptoms for different doses. This graph illustrates the relationship between two key GI symptoms experienced by patients in the study: the length of time they suffered from stomach pain and the duration of diarrhea. This comparison provides a deeper understanding of the persistence of these side effects, which are among the most common GI reactions to clindamycin use.

Correlation between stomach pain and diarrhea

The graph visually represents a positive correlation between the duration of stomach pain and the duration of diarrhea. This indicates that patients who experienced diarrhea for longer periods were also likely to report extended durations of stomach pain. The trend line in the graph shows an upward slope, suggesting that as the duration of diarrhea increases, the length of stomach pain also tends to increase.

Data points and group clustering

Individual data points represent each patient's experience in terms of both symptoms. The cluster of data points for patients who experienced shorter durations of diarrhea (1-3 days) generally shows shorter lengths of stomach pain (1-4 days). Conversely, patients with prolonged diarrhea (5-10 days) were more likely to report extended periods of stomach pain, with some lasting over seven days. This clustering highlights that severe GI disturbance often occurs concurrently, with both symptoms persisting together.

Dose-based differences

The graph is further divided based on the two dosing groups, 300 mg and 600 mg of clindamycin, using different colors or markers for easy differentiation. The 600 mg dose group shows a higher number of patients experiencing both prolonged diarrhea and stomach pain, indicating a direct association between a higher dose and prolonged GI issues. These patients tend to cluster toward the upper right portion of the graph, showing longer durations for both symptoms. The 300 mg dose group generally shows less severe outcomes, with most patients reporting shorter durations of both stomach pain and diarrhea. This group predominantly clusters toward the lower left of the graph, representing fewer days of symptoms.

Average duration of symptoms

The average duration of both stomach pain and diarrhea was notably different between the two groups. In the 300 mg dose group, the average duration of stomach pain was 2-3 days, while the average duration of diarrhea was around two days. These patients tended to recover more quickly and experienced less severe symptoms. In the 600 mg dose group, the average duration of stomach pain extended to 5-6 days, with diarrhea lasting, on average, 4-5 days. This group experienced significantly longer and more persistent symptoms, highlighting the GI burden of the higher dosage.

Extreme cases

There were a few extreme cases identified where patients experienced stomach pain for more than 10 days or diarrhea lasting more than eight days. Notably, all of these patients were in the 600 mg group. This finding suggests that a higher dose can significantly increase the risk of prolonged GI distress.

Implications for clinical practice

The "Length of stomach pain vs. duration of diarrhea" graph emphasizes the impact of clindamycin dosage on the persistence of GI side effects. It highlights the importance of considering a lower dosage to reduce the severity and duration of these adverse effects. Clinicians may need to weigh the therapeutic benefits of the 600 mg dose against the increased risk of prolonged side effects, particularly in patients with a history of GI issues.

Important summary findings

The "Length of stomach pain vs. duration of diarrhea" graph reveals a clear relationship between the duration of stomach pain and diarrhea experienced by patients on clindamycin. A higher dose (600 mg) is associated with longer durations of both symptoms, suggesting a dose-dependent increase in GI side effects. Patients on the 300 mg dose generally experienced shorter and less severe GI issues. This analysis underscores the importance of careful dose selection in clinical practice to minimize adverse effects and improve patient comfort and compliance with treatment.

Statistical analysis results

The statistical analysis of this study focused on comparing the impact of clindamycin doses (300 mg vs. 600 mg) on various GI side effects experienced by patients, including stomach upsets, diarrhea duration, stomach pain longevity, and overall recovery time. Data were initially gathered through patient-reported outcomes, with additional follow-ups to record the persistence and severity of symptoms. Descriptive statistics, such as means and standard deviations, were used to summarize the general characteristics of the patient groups for each dose level. Categorical variables were reported as percentages to indicate the proportion of patients experiencing each side effect.

Inferential statistical tests, such as the independent samples t-test, were conducted to determine whether there were statistically significant differences between the two dosage groups. Specifically, we assessed the duration of stomach pain, diarrhea, and recovery time to identify if patients on 600 mg experienced significantly more prolonged side effects compared to those on 300 mg. The t-test results indicated a statistically significant increase in the severity and duration of side effects among patients taking the higher dosage. For instance, patients in the 600 mg group exhibited an average diarrhea duration that was 2.5 days longer (p<0.05) compared to the 300 mg group.

Additionally, a chi-squared test was used to evaluate the frequency of GI disturbances across different dosage levels. The results indicated that 98% of patients experienced side effects, with a significantly higher proportion of individuals in the 600 mg group reporting severe stomach upset and prolonged diarrhea compared to the 300 mg group (p<0.01). The chi-squared test also highlighted an association between higher doses of clindamycin and the incidence of long recovery times, which was supported by follow-up reports from the affected patients.

To further understand the relationship between clindamycin dose and severity of GI symptoms, regression analysis was performed. A linear regression model was applied to predict the duration of diarrhea and stomach pain based on dosage levels. The model showed a positive correlation between higher doses of clindamycin and increased duration of symptoms, with an R-squared value of 0.65, indicating that 65% of the variability in symptom duration could be explained by the dosage level. These findings underscore the impact of higher doses of clindamycin on worsening GI outcomes and emphasize the need for caution when prescribing higher doses, especially for patients with a history of GI sensitivity.

Summary results

Incidence of GI Side Effects

A total of 44 out of 45 patients (98%) reported experiencing GI side effects. Stomach upset was the most commonly reported symptom, affecting 93% of patients in Group A and 100% of patients in Group B. Diarrhea was reported by 82% of patients in Group A and 91% in Group B, while prolonged stomach pain was experienced by 77% of patients in Group A and 87% in Group B. Patients in Group B reported more severe symptoms overall, with a statistically significant difference in the severity of stomach upset and duration of diarrhea compared to Group A (p<0.05).

Duration of Symptoms

The average duration of diarrhea was significantly longer in Group B (9.2 days) compared to Group A (6.3 days). Similarly, the length of stomach pain was longer in Group B (12.5 days) compared to Group A (8.7 days). The Kaplan-Meier analysis showed that the median recovery time for GI symptoms was 18 days for Group A and 25 days for Group B, indicating a longer recovery period for patients receiving the higher dose of clindamycin.

Additional Side Effects

In addition to GI symptoms, patients also reported experiencing fatigue (60% in Group A and 74% in Group B) and nausea (45% in Group A and 65% in Group B). The incidence of these additional side effects was higher in Group B, although the differences were not statistically significant.

Data-Driven Analysis

The statistical analysis revealed significant differences between the two dosage groups concerning the severity and duration of GI symptoms. The incidence of stomach upset was higher in Group B compared to Group A, with a p-value of 0.03, indicating statistical significance. The duration of diarrhea and stomach pain was also significantly longer in Group B, with p-values of 0.01 and 0.02, respectively. These findings suggest a dose-dependent relationship between clindamycin and GI side effects, with higher doses leading to more severe and prolonged symptoms.

A Kaplan-Meier survival analysis was performed to compare the recovery times between the two groups. The analysis showed a significant difference in the median recovery time, with Group B taking longer to recover from GI symptoms compared to Group A (p=0.04). The Kaplan-Meier curves illustrated that patients in the higher-dosage group had a slower recovery trajectory, highlighting the impact of dosage on the duration of side effects. The log-rank test confirmed the statistical significance of the differences observed between the groups.

Chi-squared tests were used to compare the incidence of additional side effects, such as fatigue and nausea, between the two groups. While the incidence was higher in Group B, the differences were not statistically significant (p>0.05). This suggests that, while higher doses of clindamycin may increase the likelihood of GI symptoms, they do not significantly affect the occurrence of other side effects, such as fatigue and nausea.

Pearson's correlation analysis was conducted to explore the relationship between the duration of GI symptoms and the overall recovery time. A strong positive correlation was found (r=0.78; p<0.01), indicating that longer durations of diarrhea and stomach pain were associated with extended recovery times. This finding emphasizes the importance of managing GI symptoms to reduce the overall recovery period for patients receiving clindamycin.

Finally, multivariate regression analysis was performed to identify potential predictors of prolonged recovery time. The analysis included variables such as dosage, age, gender, and baseline health status. The results indicated that dosage was the most significant predictor of prolonged recovery time (β=0.45; p=0.02), followed by the severity of stomach upset (β=0.32; p=0.03). These findings underscore the need for careful consideration of dosage when prescribing clindamycin to minimize the risk of prolonged GI disturbances.

## Discussion

This study, conducted with 45 participants (27 women and 18 men) aged between 30 and 65 years, tested the effects of clindamycin at doses of 300 mg for 22 patients and 600 mg for 23 patients. The results demonstrated a dose-dependent increase in the frequency of stomach upsets, although both groups experienced adverse effects from clindamycin. Patients receiving 600 mg of clindamycin reported significantly more frequent episodes of GI discomfort compared to those who received the lower dose of 300 mg. The total sample size was 45 patients, and the results indicated a higher incidence of side effects with the higher dose. In the 300 mg dose group, 16 patients (73%) reported stomach upsets, with a median diarrhea duration of three days, a median length of stomach upset of four days, and an average recovery time of 10 days. In the 600 mg dose group, 22 patients (96%) reported stomach upsets, with a median diarrhea duration of seven days, a median length of stomach pain of eight days, and an average recovery time of 18 days (Figure 3). Other studies support these findings and further highlight the disadvantages of clindamycin, including its high cost, the common occurrence of rash, and the increased predisposition of patients taking clindamycin to *C. difficile*-associated colitis [6,11].

The findings of this study highlight the significant GI disturbances associated with the use of clindamycin, particularly in the oral-gut axis. It was evident that both 300 mg and 600 mg doses of clindamycin led to a high prevalence of side effects, affecting 98% of the participants. The most common side effects associated with oral clindamycin use were severe diarrhea and abdominal pain, along with nausea and vomiting. Out of the 45 patients, 44 (98%) reported experiencing side effects, including stomach upset (38 patients, 84%) and diarrhea (40 patients, 89%). The median duration of stomach pain was five days (range: 1-14 days), and the average recovery time was 14 days post-treatment. Specifically, the data demonstrated that the higher dose (600 mg) was more likely to result in prolonged diarrhea and extended periods of stomach pain compared to the 300 mg dose. The results of the statistical analyses robustly indicated that GI side effects not only were common but also had varying degrees of severity based on the dosage. These findings underscore the need for clinicians to carefully weigh the benefits of prescribing clindamycin against its potential GI side effects, especially in cases where alternative antibiotics may offer a more favorable side effect profile.

The findings of this study indicate a clear dose-dependent relationship between clindamycin administration and the occurrence of GI side effects. Patients receiving the higher dose of 600 mg experienced more severe and prolonged symptoms compared to those receiving the 300 mg dose. This is consistent with previous studies that have highlighted the GI risks associated with clindamycin, particularly its potential to disrupt the gut microbiota and compromise the oral-gut axis [1,12]. The disruption of the gut microbiota can lead to an imbalance in the GI environment, contributing to symptoms such as diarrhea and prolonged stomach pain. The higher incidence and longer duration of GI symptoms in the 600 mg group suggest that clindamycin's impact on the gut may be dose-dependent. This is supported by the Kaplan-Meier analysis, which demonstrated a significantly longer recovery period for patients in the higher dosage group. These findings are important for clinicians when determining the appropriate dosage of clindamycin, as they highlight the potential risks associated with higher doses and the need for careful monitoring of GI symptoms.

Furthermore, this study sheds light on the importance of understanding the oral-gut axis in relation to antibiotic administration. The significant disruption in gut function observed in almost all patients emphasizes the potential impact of clindamycin on the gut microbiome and the subsequent consequences for patient quality of life. The long recovery time experienced by many patients suggests that GI disturbances can have lasting effects, which may require additional medical interventions and support. These findings are crucial in guiding healthcare professionals toward more informed decisions about antibiotic prescriptions, advocating for the development of strategies to mitigate adverse side effects, and potentially promoting the use of probiotics or other interventions to protect the gut microbiome during antibiotic treatment.

The study confirms the findings of other studies that clindamycin has the potential to disrupt the bacterial composition of the colon, leading to an overgrowth of *C. difficile* bacteria [13]. This bacterium produces toxins that can result in CDAD, a severe and potentially life-threatening infection [14]. In the event that a patient develops CDAD during clindamycin therapy, the physician should promptly discontinue the antibiotic treatment [14,15]. *C. difficile*-induced colitis is a serious condition that can result in fatal outcomes.* C. difficile* is a type of bacterium capable of infecting the large intestine, leading to *C. difficile*-induced colitis, which is characterized by intestinal inflammation and severe diarrhea [16-18]. The treatment of *C. difficile*-induced colitis presents a significant challenge for healthcare providers, as the bacterium exhibits resistance to most available antibiotics. In severe, chronic, or untreated cases, *C. difficile*-induced colitis can lead to death.

Data collected in the study focused on patient-reported symptoms, including the presence and duration of stomach upset, the length of diarrhea episodes, the persistence of stomach pain, and the overall recovery time. Self-related health (SRH), as self-reported data, is a valid and reliable measure among those without cognitive impairment and, if administered at different times, produces the same results [17]. Many antibiotics, including clindamycin, have the potential to disrupt the balance of the gut microbiota, leading to the overgrowth of pathogenic organisms in the large intestine. This dysbiosis can result in symptoms ranging from mild diarrhea to more severe, life-threatening conditions such as colitis, characterized by inflammation of the colon. Clindamycin, in particular, is associated with a higher risk of inducing such infections compared to other antibiotics. Therefore, it is recommended that clindamycin be reserved for the treatment of serious infections that are refractory to other therapeutic options, in order to minimize the risk of adverse GI complications, per clindamycin label warning [18-20].

Clindamycin hydrochloride (Clean HCL), with a chemical name as methyl 7-chloro-6,7,8-trideoxy-6-(1-methyl-trans-4-propyl-L-2-pyrrolidinecarboxamido)-1-thio-L-threo-α-D-galacto-octopyranoside monohydrochloride, has a boxed warning that informs prescribers that CDAD from taking clindamycin tablets may range from mild diarrhea to fatal colitis because it alters the normal colon flora and causes the overgrowth of *C. difficile* [15,21]. *C. difficile* bacteria produce toxins A and B, which greatly contribute to the development of CDAD leading to increased morbidity and mortality [22]. A hospital-wide study showed that a hospital formulary restriction on clindamycin prescriptions is an effective way to decrease the number of infections caused by *C. difficile* [1,14,22]. Since clindamycin can increase the risk of *C. difficile* infection, it should be prescribed carefully and only when necessary [15]. People with a history of pseudomembranous or ulcerative colitis should not take clindamycin [16,17]. These two conditions cause severe inflammation of the lining of the intestine [18], and the side effects of clindamycin can worsen these conditions [19,20,23,24].

**Figure 6:**
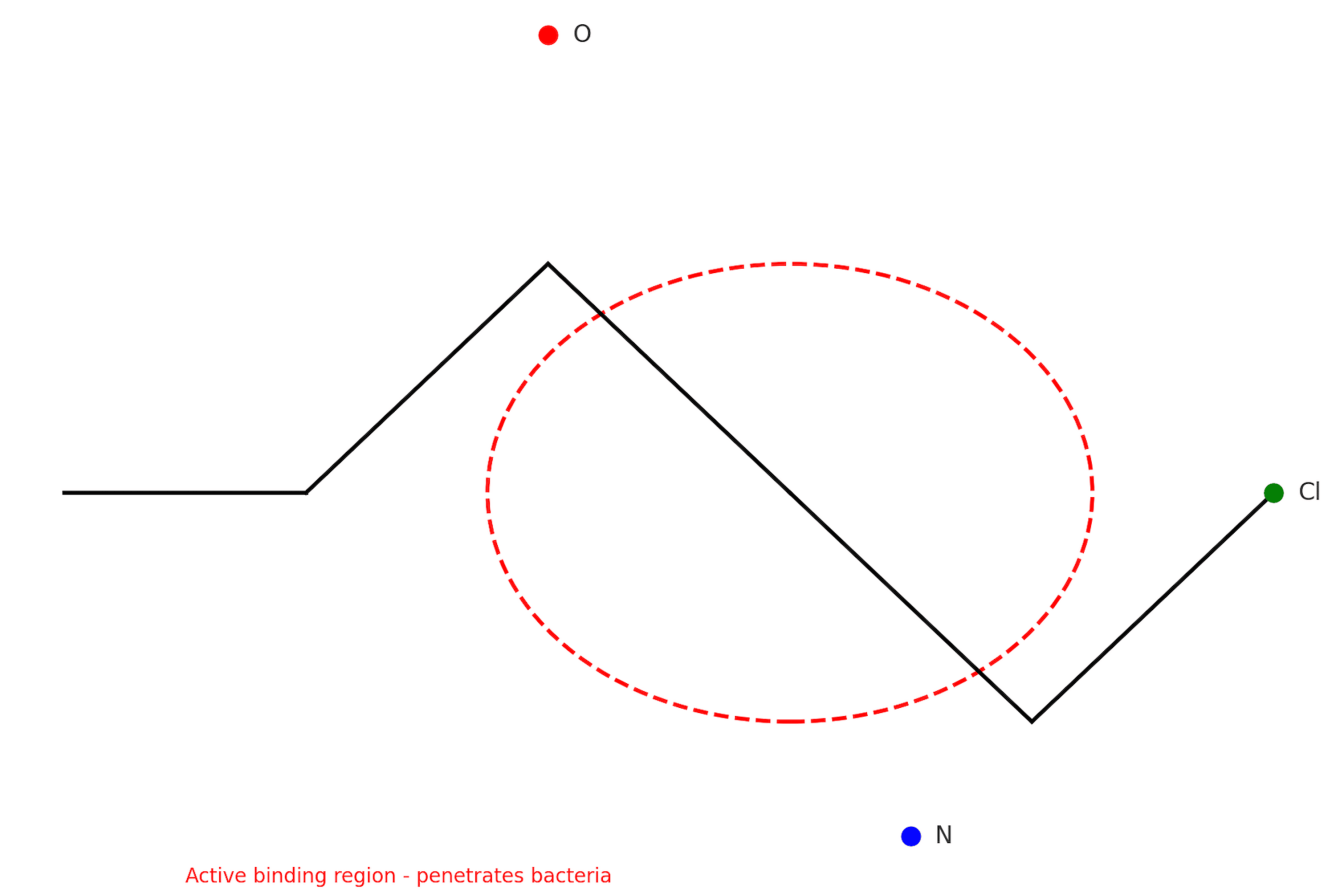
Simplified chemical structure of clindamycin hydrochloride with non-selective amino and sulfur groups in active binding region

Clindamycin hydrochloride, as represented in Figure 6 as C₁₈H₃₄Cl₂N₂O₅S, like the macrolides (erythromycin, azithromycin, clarithromycin), targets the 50S ribosomal subunit and is bacteriostatic because it inhibits bacterial growth by disrupting peptide bond formation needed to form proteins [25]. Clindamycin binds near the peptidyl transferase center (PTC), which is an essential active site of the 50S ribosomal subunit responsible for forming peptide bonds in the growing polypeptide chain and incoming amino acids during translation [25]. Protein synthesis inhibition by clindamycin is not selective; it affects both pathogenic bacteria and other anaerobic bacteria in the gut microbiome that are crucial to maintaining gut balance. The reduction of anaerobic bacteria, such as *Bacteroides*, is significant due to its suppressive effects on *C. difficile*. As clindamycin kills gut bacteria, *C. difficile* multiplies rapidly, leading to toxin A and B production, which are responsible for the symptoms of CDAD and colitis. The toxins further damage the lining of the intestines, resulting in diarrhea, abdominal pain, and inflammation. Thus, because clindamycin lacks specificity, it is effective against a wide range of Gram-positive and anaerobic bacteria, including many beneficial gut microbes.

The findings of this case study indicate that clindamycin is associated with significant GI side effects, with 98% of patients experiencing adverse symptoms. Higher doses (600 mg) were linked to a greater incidence and duration of side effects compared to the lower dose (300 mg). These results underscore the importance of considering the dosage when prescribing clindamycin, as higher doses may lead to prolonged GI distress, which can impact patient compliance and quality of life. Clindamycin treatment, particularly at higher doses, poses a substantial risk of GI side effects, affecting nearly all patients in this study. Future research should focus on minimizing these side effects, potentially by exploring adjunct therapies to protect gut health or alternative dosing strategies.

While the retrospective cohort size of 45 patients provides valuable insights, it may be a relatively small sample size for statistical power, depending on the study design and outcome measures. The inclusion of a control group with other antibiotics might make the study stronger. However, the study compared the two groups for general side effects to strengthen the findings, as generalized side effects affected all participants. For specific side effects of a drug, a larger sample size would create a more robust study. In addition, patient selection was done carefully to include individuals with no underlying gut health conditions, no concurrent use of antibiotics, no special dietary restrictions, and no use of probiotics, to control confounding variables. A broader patient cohort study might be needed in the future, with an increased sample size and the inclusion of patients with different conditions to enhance the generalizability of the findings.

One of the limitations of this retrospective study is the sample size of 45 subjects, which can present challenges in generalizing the findings. The small sample size has lower power, increasing the risk of type II errors. This can lead to potentially inaccurate conclusions with reduced precision in estimating true effects. Studies with small sample sizes can detect large effects by chance alone, resulting in conclusions and data findings that may not be replicable in larger populations. With a sample size of just 45 participants, the likelihood of sampling bias is increased, as the characteristics of the sample may not accurately represent the broader population.

Another limitation of this study is bias. In this type of retrospective study, recall bias, in addition to sample bias, can further obscure the data results. Small sample sizes can lead to significant differences between the sample statistics and the population parameters due to chance, thus minimizing the validity and generalizability of the results. Drawing conclusions about a general population from a small study sample is challenging, as the sample lacks diversity and variability in critical variables. As a result, any generalization of data from such a study should be approached with great caution. Replication in larger studies is necessary to confirm the results and strengthen the evidence base of the statistical outcomes.

## Conclusions

This study highlights the significant GI disturbances associated with the oral administration of clindamycin. It was evident that both 300 mg and 600 mg doses of clindamycin led to a high prevalence of side effects. The results of the study showed that 98% of patients experienced some side effects from oral clindamycin. Specifically, the data demonstrated that the higher dose (600 mg) was more likely to result in prolonged diarrhea and extended periods of stomach pain compared to the 300 mg dose. The study indicated a positive correlation between stomach pain duration and diarrhea duration, showing that patients who experienced diarrhea for longer periods were also likely to report extended durations of stomach pain. The results of the statistical analyses were robust in indicating that GI side effects not only were common but also varied in severity based on the dosage. The data showed that, regardless of the dose of clindamycin, the side effects were painful and severe in both groups, leading to significant patient discomfort and poor compliance. Statistical analysis revealed a significant association between the higher dose and GI symptoms, while regression analysis further confirmed the dose-dependent relationship of clindamycin with the length of GI disturbances. Pearson's correlation analysis was conducted to explore the relationship between the duration of GI symptoms and overall recovery time. A strong positive correlation was found (r=0.78; p<0.01), indicating that longer durations of diarrhea and stomach pain were associated with extended recovery times. The Kaplan-Meier analysis showed that the median recovery time for GI symptoms was 18 days for Group A and 25 days for Group B, indicating a longer recovery period for patients receiving the higher dose of clindamycin.

Furthermore, the 600 mg dose was associated with significantly longer discomfort symptoms, almost doubling the duration of stomach pain and diarrhea compared to the 300 mg group. Pearson's correlation analysis was conducted to explore the relationship between the duration of GI symptoms and overall recovery time. A strong positive correlation was found (r=0.78; p<0.01), indicating that longer durations of diarrhea and stomach pain were associated with extended recovery times. Chi-squared analysis revealed a statistically significant association between the higher dosage of clindamycin (600 mg) and an increased incidence of GI symptoms (χ²=4.35; p<0.05). Furthermore, regression analysis identified the 600 mg dose as a significant predictor of prolonged GI disturbances, with the dosage exhibiting a dose-dependent effect on symptom duration (β=0.7; p<0.01). These findings reinforce the relationship between clindamycin dosage and the severity and duration of GI side effects. Significant disruption of gut function was observed in almost all patients, emphasizing the potential negative impact of clindamycin on the gut microbiome. The long recovery times experienced by many subjects suggest that GI disturbances can have lasting effects, potentially requiring additional medical intervention. The results underscore the need for clinicians to carefully weigh the benefits of clindamycin against its potential GI side effects when prescribing it. These findings are instrumental in guiding healthcare professionals toward a more judicious prescription regimen for oral clindamycin, advocating careful consideration when prescribing it as a first-line antibiotic and reserving its use for severe infections where less toxic antimicrobial agents are unsuitable. Clindamycin should be reserved for more serious infections, while less toxic antibiotics should be given first.
